# Reorienting programme budgeting and marginal analysis (PBMA) towards disinvestment

**DOI:** 10.1186/1472-6963-10-288

**Published:** 2010-10-14

**Authors:** Duncan Mortimer

**Affiliations:** 1 Centre for Health Economics, Faculty of Business & Economics, Monash University, Melbourne, Australia

## Abstract

**Background:**

Remarkable progress has been made over the past 40 years in developing rational, evidence-based mechanisms for the allocation of health resources. Much of this progress has centred on mechanisms for commissioning new medical devices and pharmaceuticals. The attention of fund-managers and policy-makers is only now turning towards development of mechanisms for decommissioning, disinvesting or redeploying resources from currently funded interventions. While Programme Budgeting and Marginal Analysis would seem well-suited to this purpose, past applications include both successes and failures in achieving disinvestment and resource release.

**Discussion:**

Drawing on recent successes/failures in achieving disinvestment and resource release via PBMA, this paper identifies four barriers/enablers to disinvestment via PBMA: (i) specification of the budget constraint, (ii) scope of the programme budget, (iii) composition and role of the advisory group, and (iv) incentives for/against contributing to a 'shift list' of options for disinvestment and resource release. A number of modifications to the PBMA process are then proposed with the aim of reorienting PBMA towards disinvestment.

**Summary:**

The reoriented model is differentiated by four features: (i) hard budget constraint with budgetary pressure; (ii) programme budgets with broad scope but specific investment proposals linked to disinvestment proposals with similar input requirements; (iii) advisory/working groups that include equal representation of sectional interests plus additional members with responsibility for advocating in favour of disinvestment, (iv) 'shift lists' populated and developed prior to 'wish lists' and investment proposals linked to disinvestment proposals within a relatively narrow budget area. While the argument and evidence presented here suggest that the reoriented model will facilitate disinvestment and resource release, this remains an empirical question. Likewise, further research will be required to determine whether or not the re-oriented model sacrifices feasibility and acceptability to obtain its hypothesised greater emphasis on disinvestment.

## Background

Over the past 40 years, academics, evaluators and policy-makers have made quite remarkable progress in the development and application of rational, evidence-based mechanisms for the allocation of health resources. While much of this progress has centred on mechanisms for commissioning new medical devices and pharmaceuticals, attention is increasingly turning towards development of mechanisms for decommissioning, disinvesting or redeploying resources from currently funded interventions. At the macro level, England's House of Commons Health Committee has recommended that NICE give greater emphasis to identifying interventions that offer poor value for money and that might therefore be suitable candidates for disinvestment [1-3]. Australia's Medicare Benefits (MBS) Quality Framework [4] recently implemented a new process for review of existing MBS items "with the aim of identifying and evaluating MBS services which are potentially unsafe, ineffective, or inappropriately used" where delisting or amendment of the item or fee might be appropriate. Similar concerns have been recognised in technology assessment guidelines and policy statements by regional health authorities and health technology assessment agencies in Spain, by the Scottish Health Technologies Group, and by the Ontario Health Technology Advisory Committee [5]. Several national and regional agencies have taken the further step of developing and implementing tools for identifying and prioritising options for disinvestment [4-9]. At the micro level, providers and local fund-holders facing increasingly modest year-on-year growth in their budgets are piloting a range of mechanisms that might facilitate cost containment and/or redeployment of resources from currently funded interventions [9-12]. Programme Budgeting and Marginal Analysis or PBMA [13] has been suggested as one such mechanism for achieving disinvestment [12,14].

Since the first health sector application of PBMA in the early 1970s, the method has been applied in regional health services, hospital networks, individual hospitals and hospital units [13,15]. For those unfamiliar with PBMA, explanations of the underlying principles and step by step accounts of the process are available elsewhere [16,17]. Where such exercises have failed to translate 'wish lists' into action; typically, this is a consequence of a failure to release resources from elsewhere in the programme budget. The broader difficulties of disinvestment have been previously described and will not be revisited here [18,19]. Rather, the present paper identifies a number of features of PBMA that may have hampered disinvestment and resource release activities in previous applications. It is then argued that PBMA can provide a more reliable and efficient mechanism for achieving disinvestment and resource release if four features of the process can be modified to reorient PBMA towards disinvestment.

## Discussion

### Disinvestment via PBMA?

While some proponents of the approach have claimed that PBMA "necessarily links questions about investment and disinvestment of services" [20], this need not be the case during periods of rapid growth in health care expenditure. PBMA has much to offer in "maximising the benefit gained from an extra unit of resources" [17] and - rightly or wrongly - the value of PBMA during periods of rapid expenditure growth will turn primarily on its success/failure in identifying and prioritising new investments. Past applications of PBMA conducted in environments of rapid expenditure growth are therefore unlikely to assist in identifying features of PBMA that may have hampered or facilitated disinvestment and resource release. Likewise, we should not expect instruction from applications that were primarily concerned with prioritising between proposals for expansion in service provision. The discussion presented here instead focuses on recent applications of PBMA - many conducted in an environment of comparative budgetary discipline - where resource release or redeployment was explicitly identified as an objective.

Several recent applications are distinguished by their success in achieving significant resource release. For example, Mitton et al [21] describe an application of PBMA's macro equivalent: Macro Marginal Analysis or MMA, in a major regional health authority in Alberta, Canada. In this application, efficiency gains (such as shifts to generic drugs and higher patient:staff ratios) and service reductions (including rationalisation of service delivery to reduce duplication) agreed under the MMA process delivered savings of approximately 45 million Canadian dollars. Mitton et al [21] concluded that this exercise served to dispel the prevailing myth that significant resource release is not achievable via PBMA-like approaches and further commented that:

*"Although it is difficult to change clinical practices, particularly noting how entrenched health care services can become, both efficiency and service reductions can result if decision-makers have a genuine interest in improving the health of the population" *[21].

While genuine interest in pursuing social or organisational objectives (such as improving population health) rather than personal objectives (such as expanding empires or defending turf) can dictate the success or failure of the PBMA process, it is worth noting that organisational objectives vary depending upon profit/non-profit status and public/private ownership and that social objectives are not limited to population health [22]. Even where the organisational and social objectives have a single-minded regard for population health, other factors have an important part to play in facilitating disinvestment and resource release.

The available evidence also suggests that clinically meaningful resource release is achievable via PBMA-like approaches in micro applications. In applying PBMA to contain surgical waitlists in Canmore General Hospital [23], scenario analysis of adjustments to the surgical program suggested that annual savings of 11,000 Canadian dollars could be obtained from improved scheduling, fewer call-backs and shorter days ($1000/yr reduction in overtime pay) and from substitution to cheaper inputs ($5000/yr savings from cheaper sterilization methods in endoscopy plus a $5000/yr reduction in associated maintenance costs). After expansion of the surgical program to include 17 additional days of minor surgery per year (+$8925/yr), implementation of panel recommendations was expected to yield a small net resource release ($2075/yr).

While the Canmore and Calgary exercises provide some cause for optimism, PBMA has not always been so successful in achieving disinvestment and resource release. In some cases, PBMA working/advisory groups have encountered difficulties in populating a 'shift list' with options for disinvestment. For example, the advisory/working group in the Newcastle upon Tyne PCT Child and Adolescent Mental Health Services (CAMHS) exercise failed to identify a single service that might be decommissioned to free up resources for reinvestment "and therefore had to rely on future investment to fund priorities" [14]. In other cases, difficulties have arisen in progressing beyond the identification of options for disinvestment to development of business cases. After piloting PBMA in Hull PCT's diabetes programme, Baughan and Ferguson [26] observed that "identifying options for service change was relatively easy, however identifying and working up options for resource release was more challenging". Whereas fifteen of the identified options for service change in the Hull exercise either required additional resources or were initially claimed to be budget neutral, just two options were identified that had the potential to deliver resource release (changing insulin prescribing practice, and reassessing use of blood test strips) neither of which were developed into business cases. The four options that were developed into business cases were each presented as budget neutral. While implementation of budget neutral service change is not dependent on resource release from elsewhere in the programme budget, "there were cost implications... if only opportunity costs, not recognised when the business cases had been written" [26]. The exercise also identified options of 'significant marginal benefit' (not developed into business cases) that *were *dependent on resource release. Reorienting PBMA to place greater emphasis on identifying and developing options for disinvestment and resource release might - in this case - have provided a fall back in the event that claims of budget neutrality were deemed unrealistic and would have allowed greater flexibility to pursue other investments of 'significant marginal benefit'.

Even where the hurdles of identification and development of options for disinvestment have been cleared, PBMA working/advisory groups in some applications have met resistance in progressing to implementation. In applying PBMA to prioritise Norfolk Primary Care Trust's spending on mental health services [12,24], the advisory panel identified 18 disinvestment options (revised down to nine); "sufficient... to fund all the options for service change that required investment" [12]. Despite the 'sufficiency' of resource release potentially available from identified disinvestment options, participants in the Norfolk pilot were uncertain that any such options would actually be implemented. In another such example, Bohmer et al [25] applied PBMA to prioritise investments and disinvestments from respiratory diseases expenditure in New Zealand's Southern (SRHA) and Midland (MRHA) health regions. Options for investment and disinvestment were identified and prioritised in both regions but neither region implemented recommendations for disinvestment. The MRHA, for example, implemented several investments after allocating additional funds to the respiratory diseases program but made no plans for disinvestment. Interestingly, Bohmer et al reported that one of the RHAs felt 'unable to disinvest' and noted "the difficulties encountered by purchasers in making disinvestments".

### Barriers to disinvestment

While "PBMA as a local *commissioning *tool was seen by those involved as a success" (emphasis added) in many past applications [26], success in decommissioning, disinvesting or redeploying resources has sometimes proven difficult to achieve. In this section, examples are taken from past the applications - including those described above - to illustrate four barriers to disinvestment and resource release: specification of the budget constraint, scope of the programme budget, composition and role of the advisory group, and incentives for/against contributing to a 'shift list' of options for disinvestment and resource release.

#### Budgetary pressure

Past applications have demonstrated that PBMA fails to effectively link questions about investment and disinvestment of services when the budget constraint is poorly specified and/or treated as a decision variable. In the Newcastle CAMHS application, the budget constraint was poorly specified and, after receiving clarification regarding the availability of extra funds, was then treated as a decision variable. Initially, the CAMHS application proceeded as if:

*"...new service developments could only be achieved through decommissioning current services to fund reinvestment in new services, or through no cost whole system redesign. ...as the exercise progressed, service reviews conducted at other levels of CAMHS freed up potential resources for investment" *[14].

When the time came to prioritise new service developments, resources earmarked for redeployment to CAMHS were 'no longer available' due to 'political factors'. Participants in the CAMHS application were therefore, at a crucial stage of the PBMA process, under the impression that service developments would be feasible irrespective of the magnitude of resource release achieved via the PBMA process. It is not surprising then that, when disinvestment proposals were requested, 'none were forthcoming'. After it became clear that additional monies would not immediately be available to fund new service developments, there remained hope that "up our sleeves we could have funded some things (Advisory Panel member 1)" [14].

In other applications, such hopes have proven to be well-founded; with the strategy of first prioritising options for commissioning and then lobbying for relaxation of the budget constraint being employed with some success. For example, in the MRHA exercise described by Bohmer et al [25], one of the outcomes of the process was a relaxation of the budget constraint. Subsequent to prioritising options for service change, an internal working group was formed to consider the feasibility of investment and disinvestment proposals given constraints. It may be that - faced with the difficult task of implementing disinvestments - the MRHA opted for the path of least resistance. In any event, additional monies for new investments were eventually forthcoming "meaning that no disinvestment needed to occur" [25].

If the incentive to pursue disinvestments is weakened where the budget constraint is poorly specified and/or treated as a decision variable, past experience suggests that the converse is also true. While the pressure to contain surgical waitlists in the Canmore application made identification of new investments easy (additional days of surgery) and motivated population of 'shift lists', the direct link between investments and disinvestments only became apparent after running up against the budget constraint. Specifically, the primary recommendation of an additional 38 days of major surgery and 12 days of minor surgery could not be implemented given existing resources and feasible resource releases. When the panel recognised this constraint, they *were *able to match existing resources and feasible resource releases against a much more modest expansion of the surgical programme [23].

Past experience has also demonstrated that - during periods of rapid expenditure growth - commissioning new investments remains feasible *independent of any disinvestment and resource release from existing services*. Applications that have achieved significant resource release have typically done so under significant budgetary pressure (budget cuts/deficits of known magnitude) or under significant pressure on limited resources (waiting lists exceeding targets). For example, the budget for mental health services in the Norfolk PCT was cut by £2 million in the year of the PBMA pilot and faced a further cut of 8% in the year following such that "substantial disinvestments had to be identified to satisfy the planned budget cuts before any investments could be considered" [12]. While strong incentives to identify options for disinvestment and resource release were built into the PBMA process in this application (as discussed below), such incentives may not have been put in place in the absence of significant budgetary pressure.

In the Alberta MMA application, savings of some 42 million Canadian dollars were required to address the budget deficit. Mitton et al [21] suggest that "...if there was no deficit, a large amount of resources would have been available for genuine re-allocation to service growth areas". Alternatively, it may be that the substantial deficit motivated disinvestments and that participants might not have been so forthcoming with options for disinvestment in the absence of significant budgetary pressure. Given the small share of released resources allocated to new investments, it seems likely that the quite remarkable success of the Alberta application in achieving resource release was primarily attributable to significant budgetary pressure - rather than to "a genuine interest in improving the health of the population" [21].

#### Scope

Past experience suggests that the link between investments and disinvestments in the PBMA process may be weakened by the failure to specify a hard budget constraint. Failure to adequately specify the scope of the programme and the PBMA exercise can have a similar impact. When faced with a hard budget constraint, there is an incentive to shift costs by proposing service expansions for which other programme budgets would foot the bill or attempt to identify savings in other programmes with the aim of relaxing the budget constraint. While encouraging attempts at identifying potential savings in other programmes may actually increase the emphasis given to disinvestment and resource release in the PBMA process, implementation of disinvestments proposed in other areas is likely to prove challenging. In Canmore, for example, "some reasonable releases were targeted outside the surgery budget" but when it came time to prioritise options for investment and disinvestment "...panel members did not feel they had the authority to reallocate resources from other programs" [23]. Others have noted similar barriers to disinvestment wherein "decrements were considered impractical in hindsight, as they ...would also have implications for a wide range of other agencies" [27].

Past experience also suggests that simply discontinuing or restricting the range of services to be provided within a programme does not guarantee that resources will be available for redeployment to new investments. These broader difficulties of realising the savings from disinvestment are well-understood by health service administrators; to the extent that some have argued that "anything we do costs money, even if it looks like it should save some. If you try to stop people doing something that does not work they will go and do something more expensive" [28]. While the extent of such problems will depend upon the structures and processes that the provider or fund-holder has in place with respect to clinical governance, purchasing, and industrial relations; resource release may be more achievable when resources are redeployed in quite a direct manner from areas of service contraction to areas of service expansion. Or, as Twaddle and Walker [28] put it, "changes will be easier to implement if they involve the same staff, etc switching from one task to another". Where the programme budget spans a wide range of health services, delivered in different settings and serving different patient populations, direct redeployment of resources may be difficult to achieve and the breadth of the programme budget may itself prove a barrier to disinvestment.

#### Advisory group

PBMA advisory/working groups typically select members to represent stakeholder groups and to access specialist expertise necessary for the PBMA process. Advisory groups in the MHRA and SHRA applications [25], for example, included representatives of regions, consumer groups, and distinct patient populations (Maori); as well as respiratory physicians (approximately one-third of members), surgeons, general practitioners, oncologists, nursing staff, physiotherapists, hospital managers, and academics. Typically, some members (e.g. medical and surgical specialists; hospital managers) are principally selected for their expertise (e.g. understanding of treatment pathways and patient populations for relevant disease-areas; understanding of hospital costing systems and budgeting) rather than to represent the interests of stakeholders. However, past experience suggests that some 'expert' members find it difficult to set aside sectional interests:

*"Individual physicians are often more concerned with their specific area and set of patients than the overall state of health in a particular region. ...if everyone puts their head down and does not want to give up any services for the disinvestment list, health care budgets will continue to grow as they always have" *[21].

When the time comes to develop a 'wish list' of new programmes and programme expansions, there is an obvious temptation for members of working/advisory groups to 'expand empires' by proposing new investments within their own budget area. The incentive for members to contribute to a 'shift list' is much less clear-cut; and would require members to either advocate in favour of disinvestments from their own budget (and perhaps against their own personal or professional interests), or target disinvestments in other areas within the programme budget (against the interests of colleagues). This may explain why 'shift lists' are often sparsely populated (or, at least, more sparsely populated than 'wish lists').

Even where 'shift lists' have been populated with some success, the involvement of the advisory group at key stages of the PBMA process permits ample opportunity for sectional interests to defend their turf. As Elshaug and colleagues[6] put it, "some stakeholders may prefer that the status quo be maintained" (p217). Stakeholders might seek to influence selection and weighting of benefit attributes so as to cast activities slated for disinvestment in the best possible light. Likewise, stakeholders could influence the selection and weighting of evidence regarding costs and effects so as to sure-up the position of options for disinvestment (e.g. broadening inclusion criteria, equal weighting for low- and high-level evidence). Even if the ranking of an activity suggests that it is providing no additional benefit at a substantial additional cost, stakeholders have a further opportunity to advocate for the preservation of the activity prior to making any final recommendations for disinvestment.

While most would advocate on behalf of one's patients and colleagues given the opportunity, the process employed in some past applications may have amplified the natural tendency to do so. In the MHRA and SHRA, for example, advisory group members were invited to become spokespeople for expansion: i.e. "advocates *for *investment and *against *disinvestment" [25]. While Bohmer et al [25] argued that this "worked well and encouraged debate among participants", they also recognised that the failure to also nominate spokespeople for contraction: i.e. advocates *against *investment and *for *disinvestment, may have further "biased the process against disinvestment".

#### Incentives

Others have noted the weak incentive for disinvestment under the budgetary processes that operate in some health services. Mitton et al [21] characterise the culture of health care as "one of rewarding overspending, whereby individual programs get budgeted what was spent, instead of being rewarded for staying within budget". When coupled with the risk that released resources will be reclaimed by fund-holders as an efficiency or productivity dividend (rather than reallocated to fund investment options within the programme budget), it is not surprising that advisory/working groups would first seek an increase in their budget allocation or to shift costs to other programmes. For example, encouraged by the receipt of transfers from other programmes to fund deficits in previous periods, the Canmore surgical programme explored the possibility of funding service expansions using resources released from community services and long-term care [23]. While "benefits to the community from such services were deemed to be too great" to justify their disinvestment in this case, the receipt of transfers in previous periods suggest that such strategies might sometimes be effective in avoiding disinvestment and reallocation from within the programme budget.

### Reorienting PBMA towards disinvestment

#### Budgetary pressure

It does seem clear from past applications that the link between investment and disinvestment is much more direct when claw-back is required to control deficits or where health care expenditure growth has stagnated or reached steady state. PBMA asks the question: "is it possible to invest in some items on the 'wish list' by disinvesting some from the (shift) list?" [14], but *only *if new investments cannot first be funded out of expansions in the programme budget. It also seems clear that, if participants believe that additional funding can always be found from somewhere (such that planned budget cuts are not a true reflection of the likely reduction in available resources), the imperative to engage in the difficult task of disinvestment and resource release will remain weak. In such circumstances, there is a risk that PBMA will be viewed primarily as a tool for identification and prioritisation of new investments. Or, as Mitton and Donaldson [16] put it:

"...obtaining resource releases can be difficult, particularly when incentives for change such as fiscal pressure, are not present. In fact, this alone can be the reason why PBMA never really bites in some organizations".

In the past, some health service managers have been required to deliver year-on-year productivity or efficiency dividends; with fund-holders applying budgetary pressure in an attempt to move inefficient providers towards best-practice. At least two problems arise with this strategy in an environment of rapid expenditure growth. First, year-on-year budget cuts may very quickly have the effect of reducing quality and/or service levels to the point where maximisation of total patient benefit subject to total health expenditure would entail increasing rather than decreasing the programme budget. Second, fund-holders attempting to apply year-on-year budget cuts in an environment of rapid expenditure growth may encounter significant political fall-out. For many providers and fund-holders, the current operating environment is characterised by fiscal restraint rather than expenditure growth; with budgetary pressure arising out of the current economic climate rather than by artifice or design. Where disinvestment and resource release is motivated by concerns for safety and quality rather than cost containment, modifications could be made to the PBMA process that would apply budgetary pressure and provide an incentive for disinvestment without actually reducing the budget. For example, PBMA could be re-structured as a two-stage process wherein an efficiency or productivity dividend must first be delivered from disinvestment options before new investments can be considered in the second stage.

In reorienting PBMA towards disinvestment, participants should be left in no doubt as to the magnitude of the programme budget for the PBMA exercise; within which they should identify and prioritise options for investment and disinvestment. In particular, fund-holders should make clear to participants that they should not second-guess or adjust the specified budget constraint under the assumption that *ad hoc *funding will be found for high priority service expansions. Here, the assumption is made that - for many providers and fund-holders - fiscal restraint will be a feature of the operating environment for some time to come. In environments of rapid expenditure growth, further modifications to the PBMA process may be required to exert budgetary pressure and provide an incentive for disinvestment and reallocation from existing services in order to maximise total patient benefit.

#### Scope

Past experience demonstrates that the link between investments and disinvestments is weakened by failure to specify hard boundaries around the programme budget. If it is expected that resource may be taken from the oncology budget to expand activities in the respiratory budget, then the scope of the programme budget (and of the PBMA exercise) should be defined to cover both the oncology and respiratory budgets. Broadening the scope of the PBMA exercise is likely to make good sense if changes in the oncology programme result in either increased or decreased demands on respiratory services. Recall, however, direct redeployment of released resources might be difficult to achieve when areas of service contraction demand a different level, mix and quality of inputs than do areas of service expansion. There may therefore be advantages to requiring investment proposals to be linked to disinvestment proposals within a relatively narrowly defined programme budget (with relatively similar input requirements). In reorienting PBMA towards disinvestment, the programme budget should be defined broadly enough to include all *feasible *areas of service expansion and contraction but, within that programme budget, investment proposals should be linked to disinvestment proposals with relatively similar input requirements.

#### Advisory group

Past experience suggests that the composition and role of PBMA advisory/working groups often creates a bias against disinvestment. Advisory group members may be reluctant to advocate in favour of disinvestments from their own budget. Identifying resource releases from other areas of the programme budget may be relatively easy in the absence of stakeholders from those other programme areas but charging clinicians in one speciality with responsibility for formulation of a 'shift list' in another speciality may prove counter-productive. The risk of retaliation in future iterations may limit participation and the risk of creating an adversarial relationship between specialities may outweigh the benefits derived from redeployment of released resources.

Many service providers in the health sector are familiar with the difficulties of negotiating conflicting sectional interests. In one Victorian public hospital:

*"Board members were not perceived to be responsible as a group for managing the hospital, but rather were viewed as relatively autonomous members, appointed to represent sectional interests" *[29].

Worse still, strong representation of physician interests and an absence of equally strong representation of opposing interests led to physician dominance of the board. Partly to address this issue, the Victorian Health Commission Act (1980) and the Health Services Act (1988) vested greater control over the appointment of board members with the state health department and restricted the size and composition of the hospital boards. Similar changes in the composition of advisory/working groups might be employed to diminish the influence of sectional interests in the PBMA process. To date, representation of stakeholders on PBMA advisory/working groups has been considered more of a help than a hindrance; largely because representation of physicians and hospital managers has also facilitated access to specialist expertise necessary for the PBMA process and because "implementation of changes to service delivery becomes extremely difficult without acceptance and ownership of those changes" [27]. In applications conducted during periods of rapid expenditure growth (and primarily concerned with commissioning), any bias against disinvestment will receive low or zero weight in the trade-off between costs and benefits of stakeholder representation. However, quite a different trade-off might operate in applications that place greater emphasis on achieving disinvestment and resource release.

The MHRA and SHRA application described by Bohmer et al [25] suggests one possible means of reorienting PBMA towards disinvestment. If nominating "advocates *for *investment and *against *disinvestment" in the MRHA and SRHA applications introduced bias against disinvestment [25], then nominating members with the specific and sole purpose of advocating *for *disinvestment might introduce a countervailing bias. In reorienting PBMA towards disinvestment, sectional interests should be equally represented on advisory/working groups to avoid dominance of any one set of interests and any bias against disinvestment arising from representation of sectional interests should be countervailed by the appointment of advocates for disinvestment.

In addition to modifications to the *composition *of advisory/working groups, there may be scope to modify the *role *of the advisory/working group. Advisory/working group input will be crucial in describing the current programme, in populating 'wish lists' and 'shift lists', and to fill gaps in the available evidence. However, some key steps in the PBMA process might more appropriately be completed by other groups or individuals. 'Wish lists' and 'shift lists' populated by the advisory group could be supplemented by a nomination process [6,30] or using one of the several recently developed tools for identifying potential disinvestments [4-6,9]. Similarly, if the end concern is to maximise the health or welfare of the community (rather than to pursue organisational objectives), then attributes of benefit and their relative weights may be more directly accessed via citizens' juries or deliberative polling than via physicians, hospital managers or consumer representatives [31,32]. Likewise, specialist centres in health technology assessment may be better placed to identify and weight evidence regarding costs and effects than are advisory/working groups [8]. Once options for investment and disinvestment have been scored by combining community weights with evidence and expert opinion for each attribute, we might further consider whether the input of PBMA advisory/working groups is then more of a hindrance than a help in revising final rankings and arriving at recommendations. Modifications along such lines would alter the very nature of PBMA; they risk seriously damaging the feasibility and acceptability of an already difficult process and would leave fund-holders and programme managers with the difficult task of implementing recommendations that have been derived with minimal consultation. For this reason, they have not been incorporated into the reoriented model at this stage.

#### Incentives

Past experience suggests that weak incentives in the PBMA process for identifying, developing and implementing disinvestment options may be hampering disinvestment and resource release. Past experience also suggests several possible means of strengthening these incentives. In the Norwich PCT application [12,24], for example, nomination of an option for investment was only accepted if a corresponding disinvestment was nominated to the 'shift list'. While this approach resulted in a well-populated 'shift list', it did not provide sufficient incentive to progress to development of business cases for disinvestment options or onwards to implementation.

The broader disinvestment literature suggests one possible approach in encouraging participants to take the next step. To date, identification, prioritisation and implementation of disinvestment options outside of PBMA exercises has progressed largely independent of commissioning [33]. Likewise, recently developed non-PBMA processes for identification, prioritisation and implementation of disinvestment options have typically been conceived as operating independently or in parallel to commissioning processes [5,6]. For example, Ruano-Raviña and colleagues argue that

*"...just as there is a system for detection and assessment of emerging technologies, there should be another that would enable detection and assessment of technologies which may have become obsolete" *[5].

Similarly, Elshaug and colleagues [6] "...propose a *dedicated *program..." for "...identifying and assessing ineffective or non-cost-effective practices; reducing their existing use" (p269, emphasis added). The task of identifying, prioritising and implementing a 'shift list' would presumably be the primary function of a dedicated disinvestment programme and would not easily be overshadowed or sidelined by commissioning activities operating in parallel or independently. In contrast, the development of 'wish lists' in PBMA typically precedes development of 'shift lists' in a sequential process that aims to "link questions about investment and disinvestment of services" [20]. While de-coupling processes for investment and disinvestment in PBMA would remove one of its great assets, it may be possible to incorporate some of the focus and attention given to disinvestment activities in a dedicated program without weakening the link between investments and disinvestments. In reorienting PBMA towards disinvestment, the risk that disinvestment becomes sidelined or overshadowed would be much reduced by simply reversing the usual sequence so that feasible options for disinvestment and resource release are first identified and developed as business cases before considering what else might be achieved with those resources. That is to say, there can be no 'wish list' without first compiling and developing a 'shift list' of feasible options for disinvestment. At the very least, this would result in a shorter and cheaper process in the event that the barriers to disinvestment cannot be overcome; with no need to progress to a 'wish list' unless feasible options for disinvestment have first been identified and developed.

It may also be possible to provide incentives for realisation of resource release from disinvestment options (rather than merely for their identification and development). Others have made the general observation that "organisations need to agree, in principle, the ground rules for redeploying any savings made at the outset of the exercise" [26]. Mitton et al [21] suggest the general principle that only those budget areas that contributed options to the 'shift list' should subsequently be considered for re-investment of released resources. More specific guarantees might, however, be desirable given the barriers to disinvestment. For example, "financial incentives, whereby clinicians are empowered to reinvest a portion of resources released back into their services, have been shown to encourage participation" [17]. Smith et al argue along similar lines that "program managers want the ability to pair investment and disinvestment proposals - 'if we cut X, we can do Y' - and so retain any freed-up resources within their own program" [20]. Where released resources are required to meet budget deficits, guarantees might specify a minimum share of resources released from each budget area that would be available for re-deployment to safe, effective and cost-effective services within that budget area (or held over until safe, effective and cost-effective options for investment within that budget area can be identified). This type of specific guarantee has the additional advantage of consistency with recommendations made regarding the scope of the programme budget. In reorienting PBMA towards disinvestment, development of a 'shift list' of feasible options for disinvestment should precede any consideration of a 'wish list'. Investment proposals should be linked to disinvestment proposals within a relatively narrow budget area.

## Summary

At both macro and micro levels, fund-holders operating within environments of greater budgetary discipline have emphasised the need for the development and urgent application of mechanisms for disinvestment and redeployment of resources from currently funded interventions [1-3,11,12]. In response to this need, processes and tools for the identification, prioritisation and implementation of disinvestments have recently been developed and are now being utilised by health technology assessment agencies around the world [4-8]. Few of these processes explicitly "link questions about investment and disinvestment of services" [20]. Notable exceptions include recent calls to consider disinvestment of existing practice comparators whenever a replacement is approved for funding [6]. Where disinvestment processes operate independently or parallel to commissioning, there is a risk that commissioning activities will continue as if programme budgets can simply expand to accommodate demand. While PBMA is often claimed to "*necessarily *link questions about investment and disinvestment of services" [20], its variable performance in achieving meaningful resource release suggests the need for development of new mechanisms and/or modifications of the PBMA process to strengthen this link.

The discussion presented here has focused on re-orienting PBMA towards disinvestment and resource release. This attempt at distilling those aspects of PBMA likely to minimise barriers to disinvestment has drawn on the experience of many of the leading lights in the development and application of PBMA. The reoriented model is differentiated by the following features: (i) hard budget constraint together with budgetary pressure; (ii) programme budgets with broad scope but specific investment proposals linked to disinvestment proposals with similar input requirements; (iii) advisory/working groups that include equal representation of sectional interests plus additional members with responsibility for advocating in favour of disinvestment, (iv) 'shift lists' populated and developed prior to 'wish lists' and investment proposals linked to disinvestment proposals within a relatively narrow budget area.

Planning is underway to trial the re-oriented PBMA model in a Melbourne metropolitan hospital network as part of a series of projects to demonstrate alternative approaches to achieving resource release and disinvestment. It is possible that the re-oriented model sacrifices feasibility and acceptability (among clinicians and service managers) to obtain its hypothesised greater emphasis on resource release and disinvestment. This will be explicitly tested in the planned demonstration. In the meantime, debate around the re-oriented model proposed here may yield further refinements. More generally, this author would urge academics and policy analysts to contribute to the development of disinvestment policy and practice. The gradual move over the past 40 years to rational, evidence-based mechanisms for prioritizing over new drugs and devices occurred through the combined efforts of academics, analysts and policy-makers. There is a danger that political considerations will dominate disinvestment policy unless a similar effort is made now.

## List of abbreviations used

CAMHS: Child and Adolescent Mental Health Services; MMA: Macro Marginal Analysis; MRHA: Midland Region Health Authority; NICE: National Institute for Health and Clinical Excellence; PBMA: Programme Budgeting and Marginal Analysis; PCT: Primary Care Trust; SRHA: Southern Region Health Authority.

## Competing interests

The author declares that they have no competing interests.

## Authors' contributions

DM was responsible for all aspects of study conduct and design. DM drafted, finalised and approved the final manuscript.

## Pre-publication history

The pre-publication history for this paper can be accessed here:

http://www.biomedcentral.com/1472-6963/10/288/prepub
